# Inactivation of human DGAT2 by oxidative stress on cysteine residues

**DOI:** 10.1371/journal.pone.0181076

**Published:** 2017-07-11

**Authors:** Sunhee Jung, Miri Choi, Kwangman Choi, Eun Bin Kwon, Mingu Kang, Dong-eun Kim, Hyejeong Jeong, Janghwan Kim, Jong Heon Kim, Mun Ock Kim, Sang-Bae Han, Sungchan Cho

**Affiliations:** 1 Anticancer Agent Research Center, Korea Research Institute of Bioscience & Biotechnology, 30 Yeongudanji-ro, Ochang-eup, Cheongwon-gu, Cheongju-si, Chungcheongbuk-do, South Korea; 2 College of Pharmacy, Chungbuk National University, 1 Chungdae-ro Seowon-gu, Cheongju-si, Chungcheongbuk-do, South Korea; 3 Natural Medicine Research Center, Korea Research Institute of Bioscience & Biotechnology, 30 Yeongudanji-ro, Ochang-eup, Cheongwon-gu, Cheongju-si, Chungcheongbuk-do, South Korea; 4 Stem Cell Research Center, Korea Research Institute of Bioscience & Biotechnology, 125 Gwahak-ro, Yuseong-gu, Daejeon, South Korea; 5 Cancer Cell and Molecular Biology Branch, Research Institute, National Cancer Center, Ilsan-ro, Ilsandong-gu, Goyang-si, Gyeonggi-do, South Korea; 6 Department of Biomolecular Science, Korea University of Science and Technology, 217 Gajeong-ro, Daejeon, South Korea; INRA, FRANCE

## Abstract

Diacylglycerol acyltransferases (DGATs) have a crucial role in the biosynthesis of triacylglycerol (TG), the major storage form of metabolic energy in eukaryotic organisms. Even though DGAT2, one of two distinct DGATs, has a vital role in TG biosynthesis, little is known about the regulation of DGAT2 activity. In this study, we examined the role of cysteine and its oxidation in the enzymatic activity of human DGAT2 *in vitro*. Human DGAT2 activity was considerably inhibited not only by thiol-modifying reagents (NEM and IA) but also by ROS-related chemicals (H_2_O_2_ and β-lapachone), while human DGAT1 and GPAT1 were little affected. Particularly, ROS-related chemicals concomitantly induced intermolecular disulfide crosslinking of human DGAT2. Both the oxidative inactivation and disulfide crosslinking were almost completely reversed by the treatment with DTT, a disulfide-reducing agent. These results clearly demonstrated the significant role of ROS-induced intermolecular crosslinking in the inactivation of human DGAT2 and also suggested DGAT2 as a redox-sensitive regulator in TG biosynthesis.

## Introduction

Triacylglycerols (TGs), a most abundant dietary neutral lipid, are the major molecules of energy storage in most eukaryotic organisms. The excessive accumulation of TGs in tissues is one of the main causes of metabolic diseases such as obesity, fatty liver, hyperlipidemia, type II diabetes and cardiovascular diseases [1–3]. With the worldwide increase in the prevalence of these metabolic diseases, it would be of great importance to better understand the process of TG biosynthesis and the molecular mechanism of enzymes in this pathway.

Two distinct diacylglycerol acyltransferase (DGAT) enzymes, DGAT1 and DGAT2, are involved in catalyzing the last and committed step of TG biosynthesis by forming covalent bond between acyl CoA and diacylglycerol (DG) [4–6]. Both of them are localized mainly at the endoplasmic reticulum (ER), but DGAT2 is also associated with mitochondria and lipid droplets [7, 8]. Even though these two enzymes have the same catalytic activity, their protein sequences are distinct with little homology and there seem to be considerable differences in structure, tissue distribution, biochemical pathway and physiological functions [1, 9]. DGAT1 is mainly expressed in skeletal muscle, intestine and testis [5] and participates in monoacylglycerol pathway, one of the two major pathways of TG biosynthesis, using exogenous fatty acids as substrates [10, 11]. DGAT2 is highly expressed in liver and adipose tissue [6] and mediates the other pathway named as glycerol phosphate pathway by utilizing the nascent DG and *de*-*novo*-synthesized fatty acids as substrates [10, 11]. Genetic studies in mice have shown that DGAT2 has a vital role in TG biosynthesis. DGAT2 knockout mice are severely deficient in TG (~90% TG reduction) and have an impaired skin barrier function, leading to early death [12], while DGAT1 knockout mice are still viable with moderate reduction in TG (~50% TG reduction) [13, 14].

To date, limited information is available regarding the molecular mechanism and functional structure of DGAT enzymes because of the difficulties in the solubilization and purification of these membrane-anchored enzymes. The current understanding of DGAT enzymes has been acquired mainly by purifying membrane fractions from cells [15, 16] and analyzing chemical modification of the proteins [17–20]. Among diverse amino acids such as cysteine, tyrosine, lysine and histidine residues, of which can be modified, cysteine residues have been frequently examined for their chemical modification because they are highly reactive and play a significant role in protein structure, stability, localization and activity [21, 22]. For instance, free sulfhydryl groups of cysteine residues frequently located in or near the active site can be easily oxidized in response to a wide range of reactive oxygen species (ROS) generated during redox cycle, which generally regulates the catalytic activity of enzymes through reversible cysteine modification [23–26]. In this respect, there have been several efforts to examine the modification of cysteine residues in DGATs. Sauro and Strickland have shown that rat DGAT activity is susceptible to thiol-specific modifying reagents such as *N*-ethylmaleimide (NEM), *p*-Chloromercuribenzoate and iodoacetate (IA) [17]. Similar results were observed in studies of DGAT activity from the yeast *Saccharomyces cerevisiae*, fungus *Umbelopsis ramanniana* (formerly *Mortierella*) and muscle tissue of *Bos Taurus* [18–20]. These studies have suggested that cysteine residues may have a functional role in DGAT1 and/or DGAT2. However, the role of cysteine residues in human DGATs has not been reported yet.

In this study, we examined the role of cysteine residues and their oxidation in the activity of human DGATs and found that DGAT2 activity is highly susceptible to cysteine modification unlike other tested enzymes also acting in TG biosynthetic pathway. Moreover, we revealed that ROS-induced inactivation of human DGAT2 occurred mainly via its intermolecular disulfide crosslinking. Our extensive analysis clearly demonstrates the significant role of cysteine residues and their oxidative disulfide crosslinking in human DGAT2 activity.

## Materials and methods

### Materials

Hydrochloric acid (HCl), fatty acid-free bovine serum albumin (BSA), *sn*-1,2-diacylglycerol (DAG), palmitoyl-CoA, H_2_O_2,_ β-lapachone, *N*-ethylmaleimide (NEM), iodoacetate (IA) and 1,4-Dithiothreitol (DTT) were purchased from Sigma-Aldrich. Organic solvents (hexane, isopropanol, heptane, ethanol, and butanol) were obtained from DUKSAN or DAEJUNG (Republic of Korea). Diethyl ether and acetic acid were purchased from Junsei Chemical (Japan). [^14^C] glycerol, [^14^C] oleoyl coenzyme A and [^14^C] glycerol-3-phosphate were obtained from PerkinElmer. The Bac-to-Bac Baculovirus Expression System was purchased from Invitrogen (U.S.A). The detergent NP-40 was obtained from USB (USA). Anti-DGAT1 (Santa Cruz, sc-32861), anti-DGAT2 (Sigma-Aldrich, HPA013351) and anti-Magoh (Santa Cruz, sc-271405) antibodies were used for Western blot analysis.

### Extraction and preparation of enzyme sources for *in vitro* enzymatic assay

Human DGAT1, DGAT2 and glycerol-3-phosphate acyltransferase 1 (GPAT1) enzyme sources for *in vitro* enzymatic assay were prepared as described previously [27]. Human DGAT1, DGAT2 and GPAT1 proteins were produced in Sf9 insect cells by using the Bac-to-Bac Baculovirus Expression System. Sf9 insect cells were infected with recombinant baculovirus encoding each enzyme for about 3 days, harvested, and lysed by using Dounce homogenization in buffer A [10 mM Tris-HCl, 250 mM sucrose, 1 mM ethylenediamine-tetraacetic acid (EDTA), pH 7.4]. Total membrane fraction containing enriched human DGAT1 or DGAT2 was isolated by sequential centrifugations at 600 and 100,000 g and resuspended in buffer B (20 mM Tris-HCl, 250 mM sucrose, pH 7.4). For *in vitro* GPAT1 assay, the homogenate was sequentially centrifuged at 600 and 8,000 g and the collected crude mitochondrial membrane fraction was resuspended in buffer B. The protein concentration was measured using the Bradford protein quantification assay.

### Extraction-based *in vitro* DGAT activity assay

DGAT activities in total membranes, which were prepared from human DGAT2- or DGAT1-overexpressing Sf9 cells, were determined by measuring the formation of [^14^C] TG from [^14^C] oleoyl-CoA and *sn*-1,2-diacylglycerol as described previously [27]. The reaction mixture for DGAT2 assay contains 175 mM Tris-HCl (pH 7.5), 5 mM MgCl_2_ (100 mM MgCl_2_ for DGAT1 assay), 200 μM *sn*-1,2-diacylglycerol, 20 μM [^14^C] oleoly-CoA (5.5 μCi), 2 mg/ml BSA and 32 μg (50 μg for DGAT1 assay) of the membrane protein. The mixture was incubated with thiol-modifying reagents in the presence or absence of 20 mM DTT at 37°C for 20 min. Thereafter, the reaction was stopped by the addition of 1.5 ml of stop solution [2-propanol/heptane/water (80:20:2, *v/v/v*)] and vortexed with 1 ml of heptane and 0.5 ml of water. The top heptane phase was collected and washed with 2 ml alkaline ethanol solution [ethanol/0.5 N NaOH/water (50:10:40, *v/v/v*)]. The radioactivity of the top phase was quantified by liquid scintillation counting (Tri-Carb 2900TR Liquid Scintillation Analyzer, PerkinElmer).

### Extraction-based *in vitro* GPAT1 activity assay

GPAT1 activity assay was performed according to the previously reported procedure [27]. NEM or H_2_O_2_ was added to the reaction mixture containing 75 mM Tris-HCl (pH 7.5), 4 mM MgCl_2_, 8 mM NaF, 100 μM palmitoyl-CoA, 1.8 μM [^14^C] glycerol-3-phosphate and 2 mg/ml BSA. The reaction was started by adding 2 μg of mitochondrial membrane fraction to the reaction mixture in a final volume of 200 μl and then incubated at 26°C for 20 min. The reaction was stopped by mixing with 1 ml of water-saturated butanol. [^14^C] lysophosphatidic acid (LPA) was extracted by mixing the mixture with 0.5 ml of butanol-saturated water, and 0.8 ml of the organic phase was collected from it. Thereafter, it was washed once with 0.8 ml of butanol-saturated water. The radioactivity of the top phase was determined by liquid scintillation counting (Tri-Carb 2900TR Liquid Scintillation Analyzer, PerkinElmer).

### Generation of human DGAT2 mutants by site-directed mutagenesis (SDM)

Human DGAT2 clone was obtained by PCR amplification from HeLa cDNA and inserted into pcDNA3.1 vector as previously described [28]. Cysteine residues in DGAT2 were mutated to alanine residues by using QuikChange Site-Directed Mutagenesis Kit (Agilent Technologies, USA).

### TLC analysis for *de novo* TG biosynthesis in human cells

HEK293 cells (4×10^5^ cells / well) were seeded in 6 well plate and cultured in Dulbecco’s modified Eagle’s medium supplemented with 10% fetal bovine serum, 1% L-glutamate and 1% penicillin/streptomycin. Cells were transfected with plasmids expressing flag-tagged wild-type or mutant DGAT2. Forty-two hours after transfection, cells were treated with [^14^C] glycerol (0.6 μCi) and incubated for another 6 hours. Cells transfected with empty vector (pcDNA3) and treated with [^14^C] glycerol were also included as a negative control. At the end of the incubation, intracellular lipids were extracted with a mixture of hexane/isopropanol (3:2, *v/v*) and separated on a PLC silica gel plate (105744, Merck) using hexane/diethyl ether/acetic acid (80:20:1, *v/v/v*) solution as the developing solvent. The newly synthesized isotope-labeled TGs were detected by bioimaging scanner (Typhoon FLA 7000, GE Healthcare) and quantified with a bioimage analysis software (Multi gauge, Fujifilm).

### Western blot analysis of overexpressed human DGAT2 in human cells

HEK293 cells were seeded and cultured in the same condition with TLC analysis. Cells transfected with plasmids expressing flag-tagged wild-type or mutant DGAT2 were incubated for 48 hours. Thereafter, cells were harvested and subjected to SDS-PAGE followed by Western blot analysis using anti-flag and anti-Magoh antibodies. More detailed procedures of Western blot analysis were described elsewhere [29].

### Analysis of intermolecular disulfide crosslinking of DGATs

To analyze intermolecular disulfide crosslinking *in vitro*, 32 μg (DGAT2) or 50 μg (DGAT1) of total membrane extracts or 2 μg of mitochondrial membrane fraction in *in vitro* reaction mixture was treated with H_2_O_2_ or β-lapachone in the presence or absence of 20 mM DTT at 37°C for 20 min. Thereafter, 2X Novex LDS sample buffer (Invitrogen), which does not contain DTT, was added to mixtures and then heated at 100°C for 5 min. To examine intermolecular disulfide crosslinking of human DGAT2 inside cells, Huh-7 and HEK293 cells were transfected with plasmid overexpressing DGAT2 for 47 hours and then treated with H_2_O_2_ for another 1 hour. Thereafter, cells were harvested and lysed in a 1% NP-40 buffer containing 150 mM NaCl, 2 mM EDTA and 20 mM Tris-HCl (pH 7.5). After sonication of the mixtures and the subsequent centrifugation at 12,000 rpm, supernatants were mixed with 2X Novex LDS sample buffer in the presence or absence of 20 mM DTT and heated at 100°C for 5 min. Resulting mixtures were subjected to Western blot analysis using anti-DGAT1, anti-DGAT2 or anti-GPAT1 antibody.

## Results

### Thiol-specific modifying reagents selectively inhibit human DGAT2 activity *in vitro*

**Fig 1:**
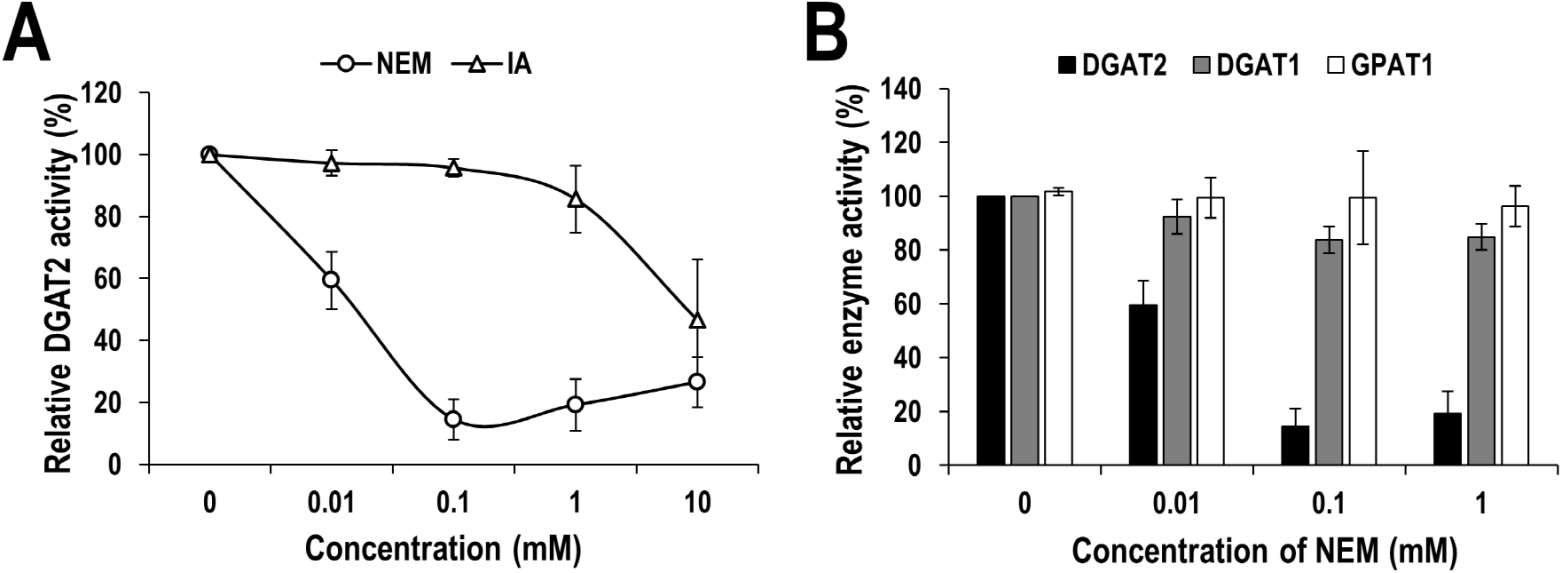
Susceptibility of human DGAT2 activity to cysteine-specific modifying reagents. (A) Membrane extracts from human DGAT2-overexpressing Sf9 insect cells were treated with indicated concentrations of NEM or IA. Human DGAT2 activity was measured by using the conventional extraction-based *in vitro* DGAT assay. The relative DGAT2 activity in percentage was calculated by setting the value from DMSO-treated sample to 100%. (B) Selective inhibitory effect of NEM on human DGAT2 activity compared to that on human DGAT1 and GPAT1. Membrane extracts from human DGAT2-, DGAT1-, or GPAT1-overexpressing Sf9 insect cells were treated with indicated concentrations of NEM or DMSO. Human DGAT1, DGAT2, and GPAT1 activity was measured by using the conventional extraction-based *in vitro* assays which are described in detail in the Materials and Methods section. The relative enzyme activity in percentage was calculated by setting the value from DMSO-treated sample to 100%. The mean values and standard deviations were determined from four independent assays.

A few previous studies have shown that DGAT activities from several different organisms are inhibited by thiol-specific modifying reagents such as NEM, *p*-chloromercuribenzoate or IA, indicating that the activity of DGATs is susceptible to the modification of cysteine residues. However, human DGATs have not yet been explored in this aspect. Therefore, in this study we tested the effect of thiol-specific modifying reagents, NEM and IA, on human DGAT1 and DGAT2 activities *in vitro*. For *in vitro* assay, membrane extract isolated from DGAT1- or DGAT2-overexpressing Sf9 insect cells was used as the enzyme sources. As a result, treatment with NEM or IA inhibited the human DGAT2 activity in a concentration-dependent manner and especially almost fully inhibited at over 0.1 mM of NEM (Fig 1A). The inhibitory effect of NEM was more efficient than IA, which is similar to that previously observed with yeast DGAT2 [20]. Intriguingly, IC_50_ of NEM on human DGAT2 was estimated as about 10 μM, which is relatively lower than that of yeast DGAT2 (~60 μM) [20]. To further know how selectively thiol-modifying reagent acts on the DGAT2, we examined the effect of NEM on human DGAT1 and GPAT1 as well. As aforementioned, DGAT1 is the isozyme of DGAT2, and GPAT1 is the enzyme acting at the first and rate-limiting step of *de novo* TG biosynthesis [30]. Unlike on DGAT2, NEM has little or marginal effect on the activities of human DGAT1 and GPAT1 even at a high concentration (Fig 1B). Consistent with our result, the resistance to thiol-modifying reagent of human GPAT1 has been already reported [31–34]. These results indicate that human DGAT2 is highly susceptible to cysteine modification unlike DGAT1 and GPAT1.

### Cysteines significantly contribute to human DGAT2 activity

**Fig 2:**
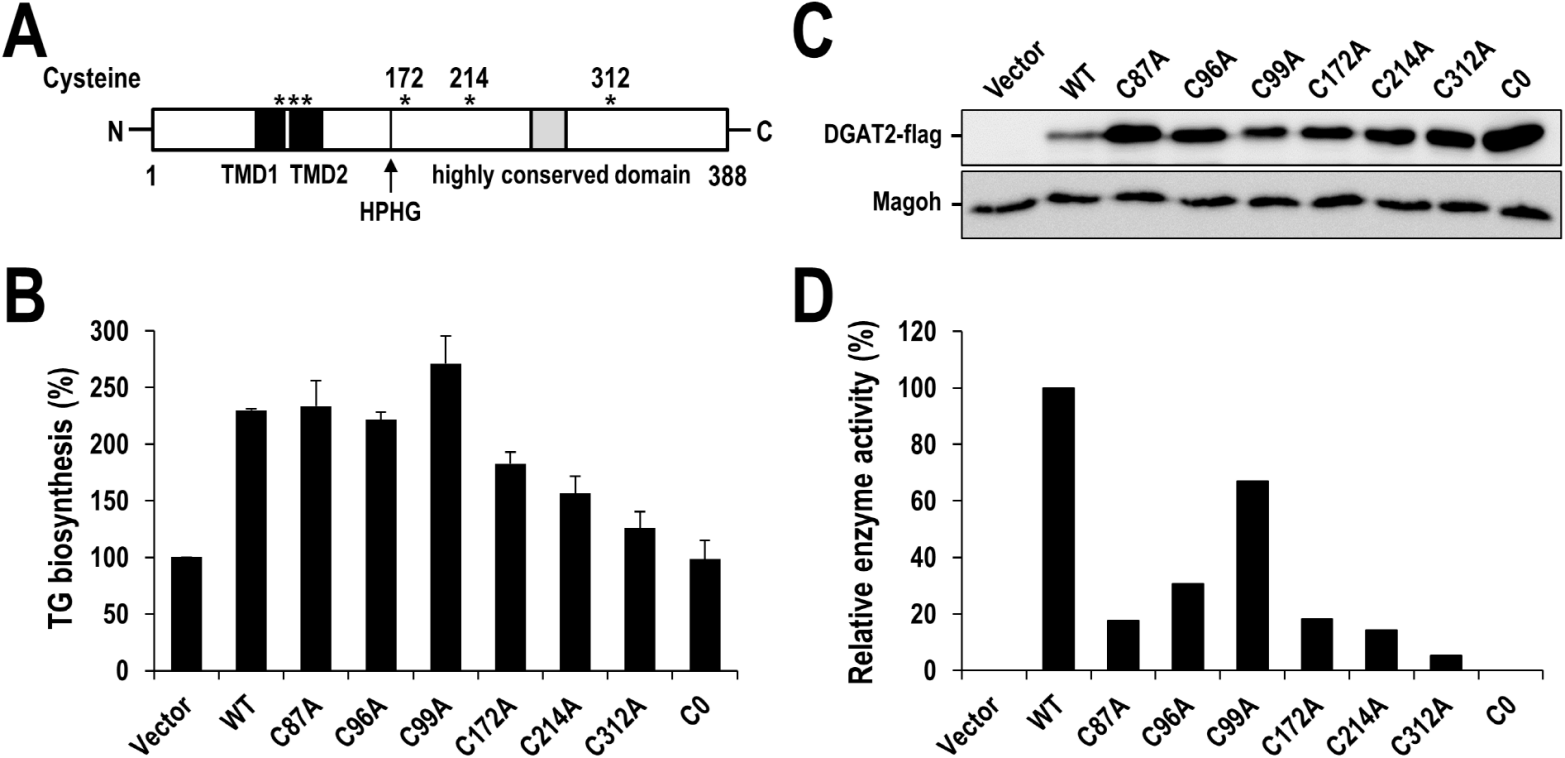
Significant role of cysteines in human DGAT2 activity. (A) The location of cysteine residues in human DGAT2 was depicted by asterisks. The transmembrane domains (TMD) and the highly conserved domain are indicated by black and grey squares, respectively. Black vertical line indicates the HPHG motif. (B) The amount of newly synthesized TG in HEK293 cells overexpressing flag-tagged wild-type, mutants (C87A, C96A, C99A, C172A, C214A and C312A) with single cysteine to alanine substitution and mutant (C0) human DGAT2 with all cysteines to alanine substitution. Wild-type and mutant human DGAT2 were overexpressed in HEK293 cells for 42 hours and incubated in the presence of [^14^C] glycerol for additional 6 hours. Newly synthesized [^14^C] TG was extracted and measured by TLC analysis. The relative TG synthesis in percentage was calculated by setting the value from pcDNA3 vector-transfected cells to 100%. The mean values and standard deviations were determined from three independent experiments. (C) The immunoblots of wild-type and mutant human DGAT2. Flag-tagged wild-type and mutant human DGAT2 were overexpressed in HEK293 cells for 48 hours. Cell extracts were harvested and subjected to Western blot analysis using anti-flag antibody. Magoh protein was examined as a loading control. (D) The normalized human DGAT2 activities of wild-type and mutant human DGAT2. Relative human DGAT2 activities were determined by dividing the amount of newly synthesized TG in panel B by the relative protein amount in panel C and S2 Fig. The normalized activity of wild-type DGAT2-transfected cells was defined as 100%.

Human DGAT2 has six cysteine residues with three of them located in the transmembrane domains and the rest of them located in C-terminal cytosolic domain (Fig 2A). We further confirmed the contribution of cysteine(s) to DGAT2 activity by generating C0 mutant that all six cysteines were mutated to alanine. Wild-type and C0 mutant of DGAT2 were expressed in HEK293 cells and *de novo* TG biosynthesis was measured by the incorporation of isotope-labeled substrate into newly synthesized TG as described in Material and Method (see the section ‘TLC analysis for *de novo* TG biosynthesis in human cells’) (Fig 2B and S1 Fig). Overexpression of wild-type DGAT2 increased the amount of cellular TG by about 130%. In contrast, cells with overexpressed C0 mutant showed TG level almost close to that of control, indicating that C0 mutant had little enzymatic activity, even though C0 proteins were expressed much more than that of wild-type (Fig 2C and S2 Fig). This result clearly demonstrated that at least one of the cysteines in human DGAT2 significantly contributes to the catalytic activity. Therefore, we further generated six mutants in which each cysteine was mutated to alanine and examined the contribution of each cysteine to the full activity of human DGAT2. Six DGAT2 mutants were individually overexpressed in HEK293 cells. Forty-eight hours after transfection, cellular TGs were measured (Fig 2B and S1 Fig) and normalized by the amount of corresponding proteins that was determined by Western blotting (Fig 2C and S2 Fig). Consequently, all six mutants showed the decreased catalytic activity compared to the wild-type, even though the extent of decrease was quite different (Fig 2D). Intriguingly, three mutants of C172A, C214A and C312A had relatively lower TG synthetic activity than other mutants such as C96A and C99A (Fig 2D). This observation can be explained, at least partly, by the fact that C172, C214 and C312 are located at C-terminal half of DGAT2 where is majorly responsible for catalytic activity. Collectively, these results support the significant role of cysteine(s) in human DGAT2 activity.

### ROS inhibits human DGAT2 activity mainly through disulfide crosslinking

**Fig 3:**
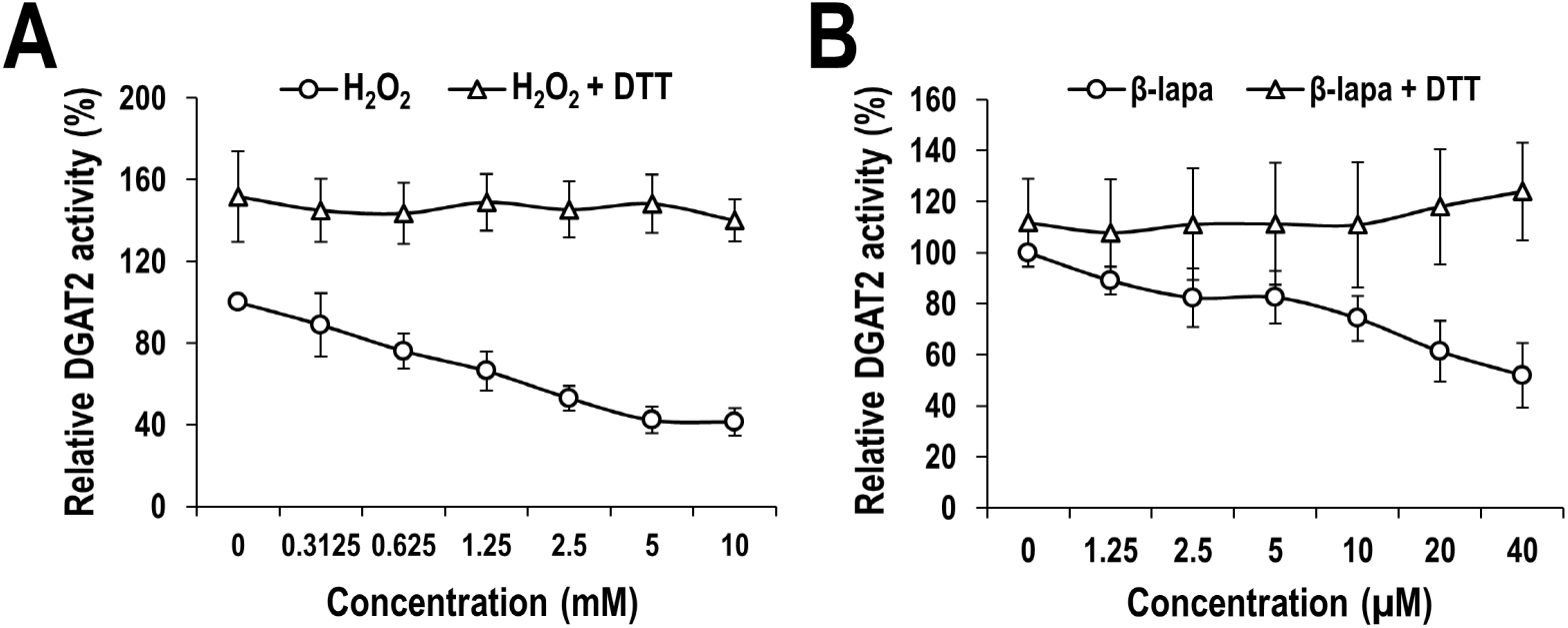
Inhibitory effect of ROS and ROS generator on human DGAT2 catalytic activity. Membrane extracts from human DGAT2-overexpressing Sf9 insect cells were treated with indicated concentrations of H_2_O_2_ (A) or β-lapachone (B) in the presence or absence of 20 mM DTT. Human DGAT2 activity was measured by using the conventional extraction-based *in vitro* assays which are described in detail in the Materials and Methods section. The activities of membrane extracts treated with PBS (instead of H_2_O_2_) or DMSO (instead of β-lapachone) in the absence of DTT were defined as 100%. The mean values and standard deviations were determined from four independent experiments.

According to our results presented above, human DGAT2 activity is inhibited by thiol-reactive reagents that generate irreversible alkylation on sulfhydryl group of cysteine (Fig 1A), and mutation of single cysteine has a negative effect on human DGAT2 activity (Fig 2B–2D). These observations prompted us to test the effect of other ROS-related agents such as hydrogen peroxide (H_2_O_2_) and β-lapachone as well. H_2_O_2_ is one of the natural ROS capable of oxidizing cysteines, and β-lapachone catalyzes the formation of superoxide anion (O_2_^-^) and H_2_O_2_ as the ROS generator of redox cycles [35, 36]. ROS can oxidize the sulfhydryl group of cysteine residue to produce sulfenic acid adducts that can either form disulfide crosslinking (-S-S-) with other cysteines or be irreversibly oxidized further to sulfinic acid moieties [37]. Particularly, disulfide crosslinking is of great importance in that it is one of the post-translational protein modifications and has the diverse role in the regulation of protein structure, stability and activity. Therefore, we examined if human DGAT2 activity is also inhibited by ROS-related agents, and disulfide crosslinking is involved in this inhibition. If that is the case, the disulfide-involved inhibitory effect can be protected by DTT, a disulfide-reducing reagent. As shown in Fig 3, *in vitro* human DGAT2 activity was considerably inhibited by the treatment with H_2_O_2_ and β-lapachone in a concentration-dependent manner. Consistent with the result in Fig 1B, human DGAT2 was more susceptible to both H_2_O_2_ and β-lapachone than human DGAT1 (S3 Fig). Moreover, the inhibitory effect of H_2_O_2_ and β-lapachone was almost completely protected by the co-treatment with DTT even at the highest concentration of them (Fig 3), indicating that ROS and ROS generator inhibited the human DGAT2 activity mainly through the formation of disulfide crosslinking. Intriguingly, treatment with DTT alone slightly increased DGAT2 activity, indicating that DGAT2 enzyme source used for this experiment contains a certain extent of disulfide crosslinking that has a negative effect on enzyme activity, which also supports the involvement of disulfide crosslinking.

### ROS and ROS generator induce intermolecular disulfide crosslinking in human DGAT2

**Fig 4:**
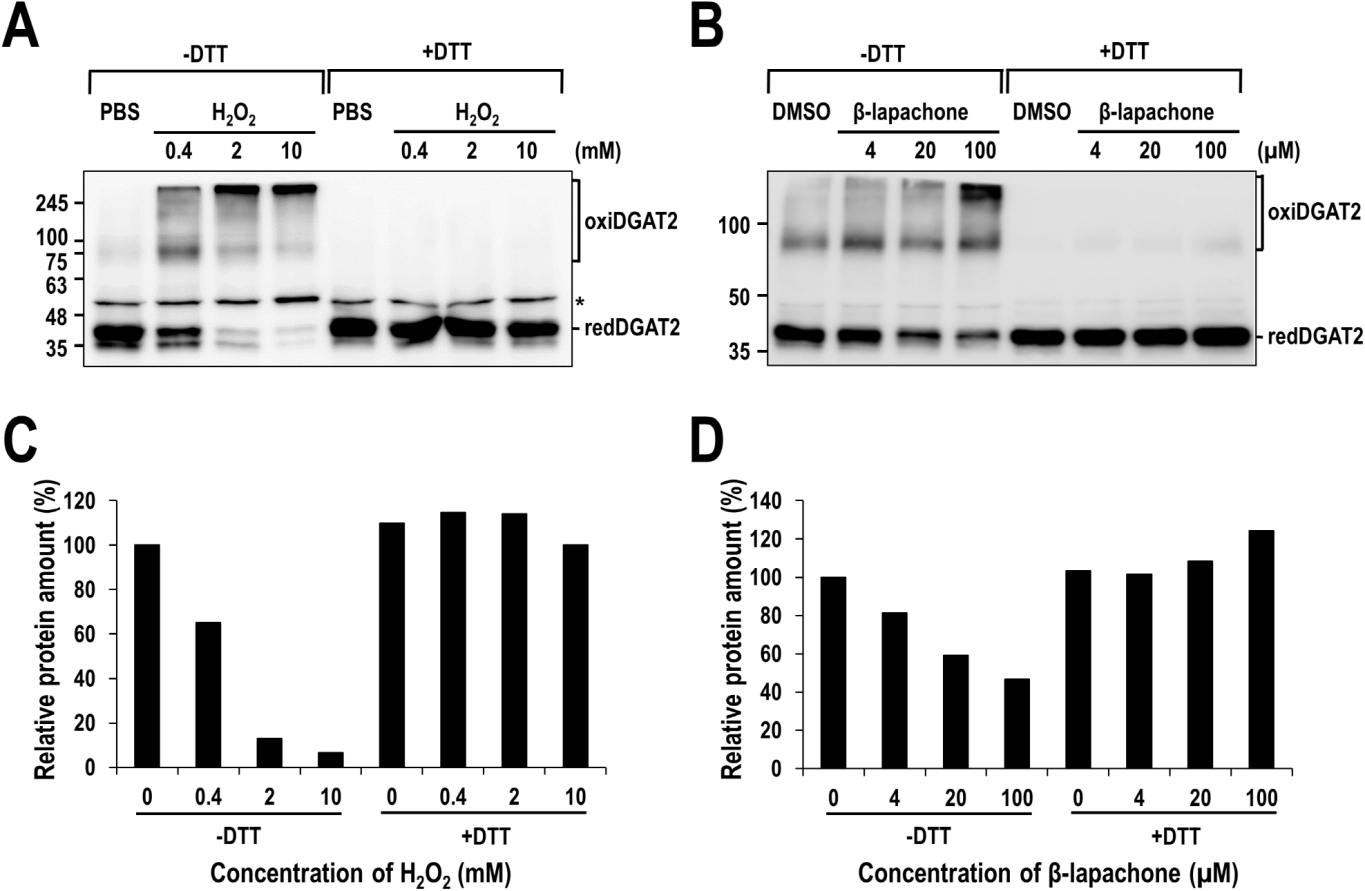
Multimeric complex of human DGAT2 formed by ROS-induced intermolecular disulfide crosslinking *in vitro*. Membrane extracts from human DGAT2-overexpressing Sf9 insect cells were treated with H_2_O_2_ (A) or β-lapachone (B) in the presence or absence of 20 mM DTT and subjected to Western blot analysis using anti-DGAT2 antibody. The amount of monomeric human DGAT2 proteins presented as redDGAT2 in (A) and (B) was quantified and the amount of relative redDGAT2 protein was calculated by setting the values from samples treated with PBS (C) or DMSO (D) to 100%. Asterisk indicates a non-specific band.

Recently, murine DGAT2 has been revealed to be part of a multimeric complex consisting of several DGAT2 subunits [8]. Especially, intermolecular disulfide crosslinking is frequently engaged in the formation of multimeric proteins and even their activity. Besides, our observation that the inhibitory effect of ROS-related chemicals on DGAT2 was fully protected by DTT strongly indicates the involvement of disulfide crosslinking. We, therefore, tested if ROS-induced intermolecular disulfide crosslinking indeed occurs in human DGAT2. To test this *in vitro*, membrane extracts isolated from human DGAT2-overexpressing Sf9 insect cells were treated with H_2_O_2_ or β-lapachone and prepared in gel electrophoresis sample buffer with or without DTT, and then analyzed by Western blotting with anti-DGAT2 antibody. As shown in Fig 4, the amount of human DGAT2 monomeric forms (~ 42 kDa) in the absence of DTT gradually decreased in a concentration-dependent manner of H_2_O_2_ or β-lapachone. Particularly, a dramatic reduction (over 90%) was observed by the treatment with H_2_O_2_, but a moderate reduction (~ 50%) by β-lapachone. Conversely, several higher molecular weight of human DGAT2 antibody-reactive forms (oxiDGAT2), corresponding in size possibly to human DGAT2 dimers (~ 80 kDa) and multimeric forms (~ 300 kDa), appeared. Unlike human DGAT2, the amount of human DGAT1 monomeric forms slightly decreased by only 20% at the highest concentration of H_2_O_2_ and were not affected by β-lapachone (S4 Fig). Strikingly, in the presence of DTT, all the higher molecular weight of human DGAT2 antibody-reactive bands were disappeared and reversed to the monomeric forms (Fig 4), indicating that ROS-induced intermolecular disulfide crosslinkings mediate the formation of multimeric protein complex involving human DGAT2. Importantly, oxidative crosslinking of human DGAT2 corresponded well to the inhibitory effect of H_2_O_2_ and β-lapachone (compare Fig 3 with Fig 4).

**Fig 5:**
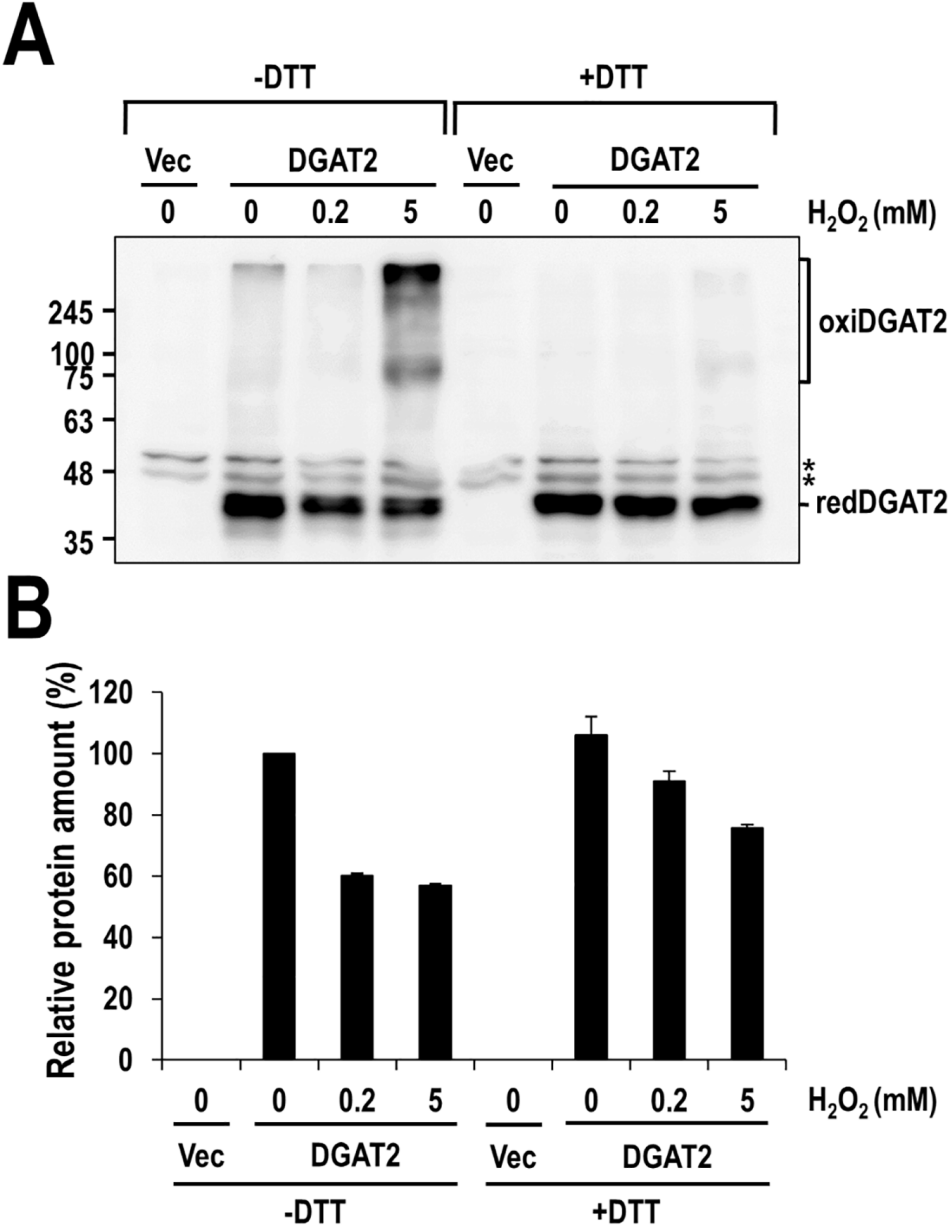
Multimeric complex of human DGAT2 formed by H_2_O_2_-induced disulfide crosslinking in human cells. Huh-7 cells were transfected with plasmid overexpressing human DGAT2 for 47 hours and further incubated with indicated concentrations of H_2_O_2_ for 1 hour. Cell extracts were harvested in a way described in Materials and Methods section and subjected to Western blot analysis using anti-DGAT2 antibody (A). The amount of monomeric human DGAT2 proteins presented as redDGAT2 in (A) was quantified and the amount of relative redDGAT2 protein was calculated by setting the values from samples treated with PBS to 100% (B). The mean values and standard deviations were determined from three independent experiments. Asterisks indicate non-specific bands.

In order to further verify the formation of the intermolecular disulfide crosslinking of human DGAT2 in a physiologically relevant environment of oxidative stress, Huh-7 cells overexpressed with human DGAT2 were treated with H_2_O_2_ and subjected to Western blot analysis as described in Materials and Methods section. As shown in Fig 5, overexpressed DGAT2 itself formed multimeric DGAT2 complexes (~ 80 and 300 kDa) at normal cells (See the lane 2). Moreover, protein bands of multimeric DGAT2 complexes became stronger by the treatment with H_2_O_2_, with a reciprocal reduction of monomeric DGAT2 band, and were disappeared in the presence of DTT (Fig 5). Similar phenomenon was also observed with DGAT2 in HEK293 cells (S6 Fig). Taken together, these results demonstrated that ROS-induced intermolecular disulfide crosslinking of human DGAT2 occurred inside human cells as well as *in vitro*.

## Discussion

In this study, we examined the role of cysteine residues and their oxidation in human DGAT2 and found that they significantly contribute to the enzymatic activity of human DGAT2, particularly, through the formation of the intermolecular disulfide crosslinking under the redox condition.

Our extensive analysis clearly demonstrates the significant contribution of cysteines to human DGAT2 activity. First, two thiol-specific alkylating reagents, NEM and IA, considerably inhibited the enzymatic activity of human DGAT2 (Fig 1A). Irreversible alkylation induced by NEM and IA might directly hinder substrate binding in or near the active site of human DGAT2 or conformational change necessary for its full activity. Intriguingly, the inhibitory effect of NEM was much stronger than that of IA, as shown by much lower IC_50_ of NEM (~ 10 μM) compared to that of IA (~ 10 mM), which is similar to that previously observed with yeast DGAT2 [20]. It might be due to that the reaction of NEM with sulfur in cysteine is much faster than that of IA [38]. Alternatively, relatively larger alkyl group formed by NEM might be more effective to cause this inhibition [20, 39]. Second, DGAT2 mutant (C0), in which all cysteines were mutated to alanine, had little TG-synthetic activity in HEK293 cells (Fig 2), strongly supporting the contribution of cysteine(s) to the activity. Further analysis with six DGAT2 mutants containing single cysteine-to-alanine mutation revealed the actual contribution of all individual cysteines to the full activity of DGAT2 (Fig 2D). Particularly, three cysteines (C172, C214 and C312), showing the lowest TG-synthetic activity with their mutants, would be noteworthy in that they are located in C-terminal half of DGAT2, especially close to two putative active sites (‘HPHG motif’ and ‘highly conserved domain’ depicted in Fig 2A) [20, 40, 41]. Further studies on the protein structure-based enzymatic activity will be required to precisely define the mode of contribution of each cysteine. Third, similar inhibition on human DGAT2 was observed by the treatment with ROS (H_2_O_2_) and ROS generator (β-lapachone), both of which could induce the oxidative modification in cysteines (Fig 3). Further analysis revealed that considerable intermolecular disulfide crosslinkings were induced *in vitro* by H_2_O_2_ and β-lapachone (Fig 4) and even in human cells by H_2_O_2_ (Fig 5 and S6 Fig). Strikingly, inhibitory effect of H_2_O_2_ and β-lapachone were completely reversed by a disulfide-reducing reagent, DTT (Fig 3), consequently demonstrating the critical role of intermolecular disulfide crosslinking in DGAT2 inactivation. This outcome was further confirmed by observing the complete conversion of intermolecularly crosslinked DGAT2 to monomeric form in the presence of DTT (Fig 4). According to a few previous reports, DGAT2 can interact with each other and is capable of forming multimeric complex [8, 42]. Besides, it can interact with monoacylglycerol acyltransferase 2 (MGAT2), fatty acid transport protein 1 (FATP1) and stearoyl-CoA desaturase 1 (SCD1), all of which function in TG biosynthetic pathway [42–44]. Especially, the interaction with SCD1 is noteworthy in that it would facilitate TG biosynthesis by efficiently relaying its product to DGAT2. Therefore, it is conceivable that human DGAT2 would be crosslinked with each other or with other interacting protein(s) in response to ROS.

One of the interesting questions is whether oxidative inactivation is specific to DGAT2 over other enzymes, particularly isozyme DGAT1, in TG biosynthesis pathway. We, therefore, applied the similar approach to human DGAT1 and GPAT1, both of which are acyltransferase like DGAT2. Intriguingly, those enzymes were marginally affected by thiol-modifying reagents, NEM, H_2_O_2_ and β-lapachone (Fig 1B and S3–S5 Figs), despite high content of cysteine residues. Human DGAT1 has 8 cysteines and most of them are located in luminal side of ER. Thus, it is a reasonable explanation that cysteines in human DGAT1 are not directly involved in its catalytic activity or are less accessible to thiol-modifying reagents. Given that ROS-related chemicals used in our studies are known to cross the intracellular membranes, cysteines, particularly one(s) involved in enzyme activity, are unlikely exposed to the surface of DGAT1 protein. This explanation can be also applied to human GPAT1, well known NEM-resistant protein [31–34], which has 17 cysteines. In addition, it should be noted that the estimated IC_50_ of NEM on human DGAT2 in our study (Fig 1A) was six folds lower than that on yeast DGAT2 in the previous report [20], indicating that human DGAT2 seems more susceptible to cysteine modification than yeast DGAT2. According to the proposed topology model, three cysteines (C172, C214 and C312) of human DGAT2 are located near two putative active sites in the cytosolic C-terminal region, while only one cysteine (C314) of yeast DGAT2 is [40, 41]. The number of cysteines located near the active site might be the reason of the difference in the susceptibilities of DGAT2 activities. Consequently, only human DGAT2 among tested enzymes working in TG biosynthesis susceptibly responded to oxidative stress mainly through the formation of intermolecular disulfide crosslinking, suggesting DGAT2 as a redox-sensitive regulator in TG-synthetic pathway.

In this study we clearly demonstrated the significance of cysteine residues in human DGAT2 activity and also suggested human DGAT2 as a redox-sensitive regulator in TG biosynthesis. Especially, ROS-induced intermolecular disulfide crosslinking was a critical event for DGAT2 inactivation. Further studies in the more physiological redox environments will make a better understanding on the regulation of human DGAT2 activity.

## Supporting information

S1 Fig TLC analysis of intracellular lipids extracted from cells overexpressed with wild-type or mutants human DGAT2. Wild-type or mutants (C87A, C96A, C99A, C172A, C214A, C312A and C0) human DGAT2 were overexpressed in HEK293 cells for 42 hours and incubated in the presence of [^14^C]-glycerol for additional 6 hours. Intracellular lipids were extracted from cells and separated on a PLC silica gel plate using hexane/diethyl ether/acetic acid (80:20:1, v/v/v) solution as the developing solvent. Radiolabeled spots were visualized by bioimaging scanner (Typhoon FLA 7000, GE Healthcare). [^14^C]-incorporated TG was indicated by the arrow.
(TIF)

S2 Fig The relative protein amount of wild type and mutant human DGAT2. The band intensity of DGAT2 proteins in Fig 2C was quantified and normalized by the corresponding band intensity of Magoh proteins. The relative protein amount was calculated by setting the value from wild-type human DGAT2 to 1. The mean values and standard deviations were determined from three independent experiments.
(TIF)

S3 Fig The effect of ROS and ROS generator on human DGAT1 catalytic activity. Membrane extracts from human DGAT1-overexpressing Sf9 insect cells were treated with indicated concentrations of H_2_O_2_ (A) or β-lapachone (B) in the presence or absence of 20 mM DTT. The activities of membrane extracts treated with PBS (instead of H_2_O_2_) or DMSO (instead of β-lapachone) in the absence of DTT were defined as 100%. The mean values and standard deviations were determined from four independent experiments.
(TIF)

S4 Fig The effect of ROS and ROS generator on intermolecular disulfide crosslinking of human DGAT1. Membrane extracts from human DGAT1-overexpressing Sf9 insect cells were treated with H_2_O_2_ (A) or β-lapachone (B) in the presence or absence of 20 mM DTT and subjected to Western blot analysis using anti-DGAT1 antibody. The amount of monomeric human DGAT1 proteins presented as redDGAT1 in (A) and (B) was quantified and relative redDGAT1 protein amount were calculated by setting the values from samples treated with PBS (C) or DMSO (D) to 100%. Asterisk indicates a non-specific band.
(TIF)

S5 Fig Resistance of human GPAT1 activity to thiol-modifying reagents. (A) The effect of NEM and H_2_O_2_ on human GPAT1 activity compared to that on human DGAT2. Membrane extracts from human DGAT2- or GPAT1-overexpressing Sf9 insect cells were treated with indicated concentrations of NEM or H_2_O_2_. Human DGAT2 and GPAT1 activities were measured by using the conventional extraction-based *in vitro* assays which are described in detail in the Materials and Method section. The relative enzyme activity in percentage was calculated by setting the value from DMSO-treated sample to 100%. The mean values and standard deviations were determined from three independent assays. (B) Membrane extracts from human GPAT1-overexpressing Sf9 insect cells were treated with β-lapachone in the presence or absence of 20 mM DTT and subjected to Western blot analysis using anti-GPAT1 antibody. Monomeric human GPAT1 proteins were presented as redGPAT1.
(TIF)

S6 Fig Multimeric complex of human DGAT2 formed by H_2_O_2_-induced disulfide crosslinking in HEK293 cells. HEK293 cells were transfected with plasmid overexpressing human DGAT2 for 47 hours and further incubated with indicated concentrations of H_2_O_2_ for 1 hour. Cell extracts were harvested in a way described in Materials and Methods section and subjected to Western blot analysis using anti-DGAT2 antibody (A). The amount of monomeric human DGAT2 proteins presented as redDGAT2 in (A) was quantified and the amount of relative redDGAT2 protein was calculated by setting the values from samples treated with PBS to 100% (B). The mean values and standard deviations were determined from three independent experiments. Asterisks indicates a non-specific bands.
(TIF)
