# Supplementary figures and images for: Inactivation of human DGAT2 by oxidative stress on cysteine residues
Source: PLoS One. 2017 Jul 11;12(7):e0181076. doi: 10.1371/journal.pone.0181076 (PMC5507451; doi:10.1371/journal.pone.0181076)

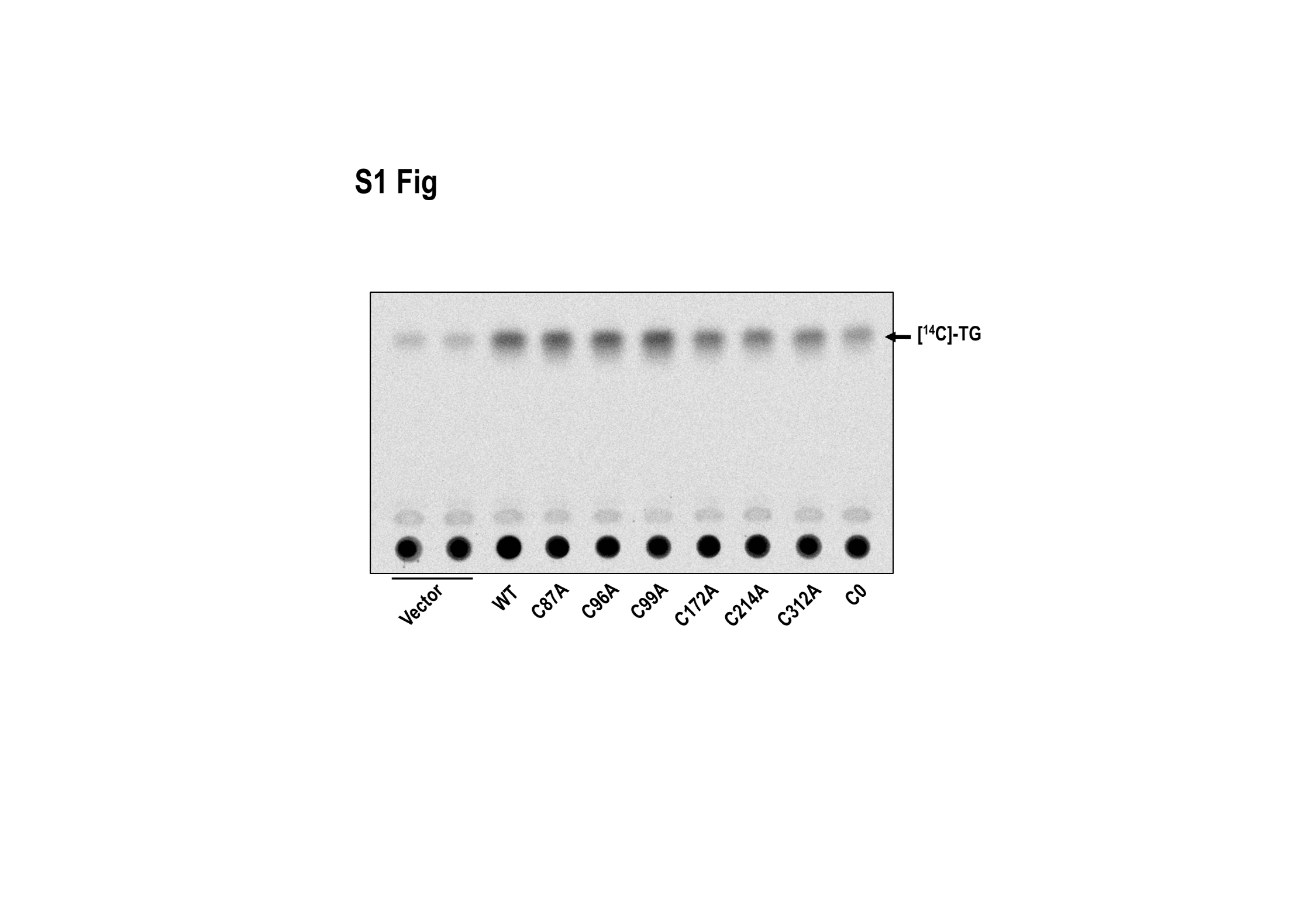

Supplement: S1 Fig — Wild-type or mutants (C87A, C96A, C99A, C172A, C214A, C312A and C0) human DGAT2 were overexpressed in HEK293 cells for 42 hours and incubated in the presence of [14C]-glycerol for additional 6 hours. Intracellular lipids were extracted from cells and separated on a PLC silica gel plate using hexane/diethyl ether/acetic acid (80:20:1, v/v/v) solution as the developing solvent. Radiolabeled spots were visualized by bioimaging scanner (Typhoon FLA 7000, GE Healthcare). [14C]-incorporated TG was indicated by the arrow. (TIF) [file pone.0181076.s001.TIF]

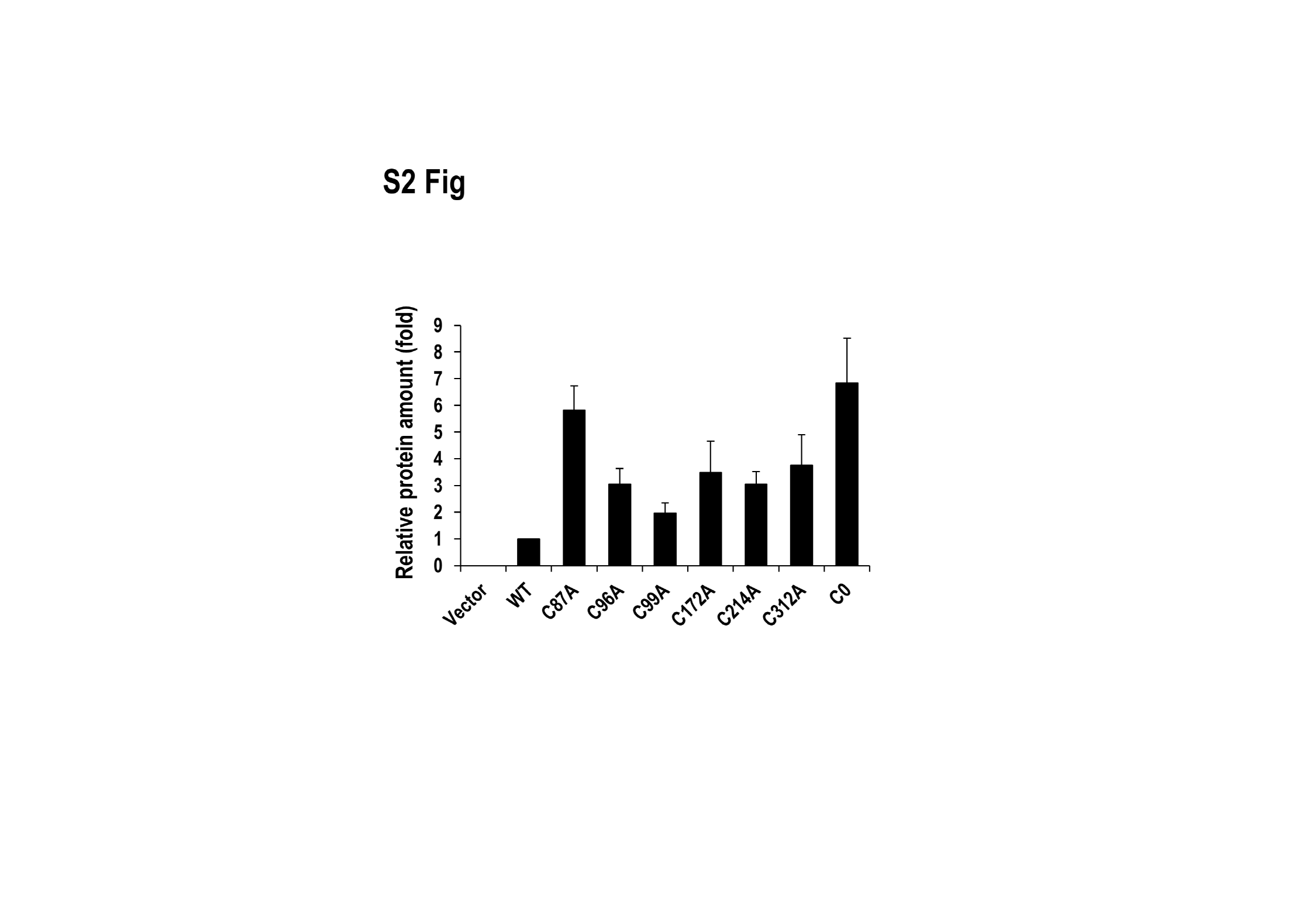

Supplement: S2 Fig — The band intensity of DGAT2 proteins in Fig 2C was quantified and normalized by the corresponding band intensity of Magoh proteins. The relative protein amount was calculated by setting the value from wild-type human DGAT2 to 1. The mean values and standard deviations were determined from three independent experiments. (TIF) [file pone.0181076.s002.TIF]

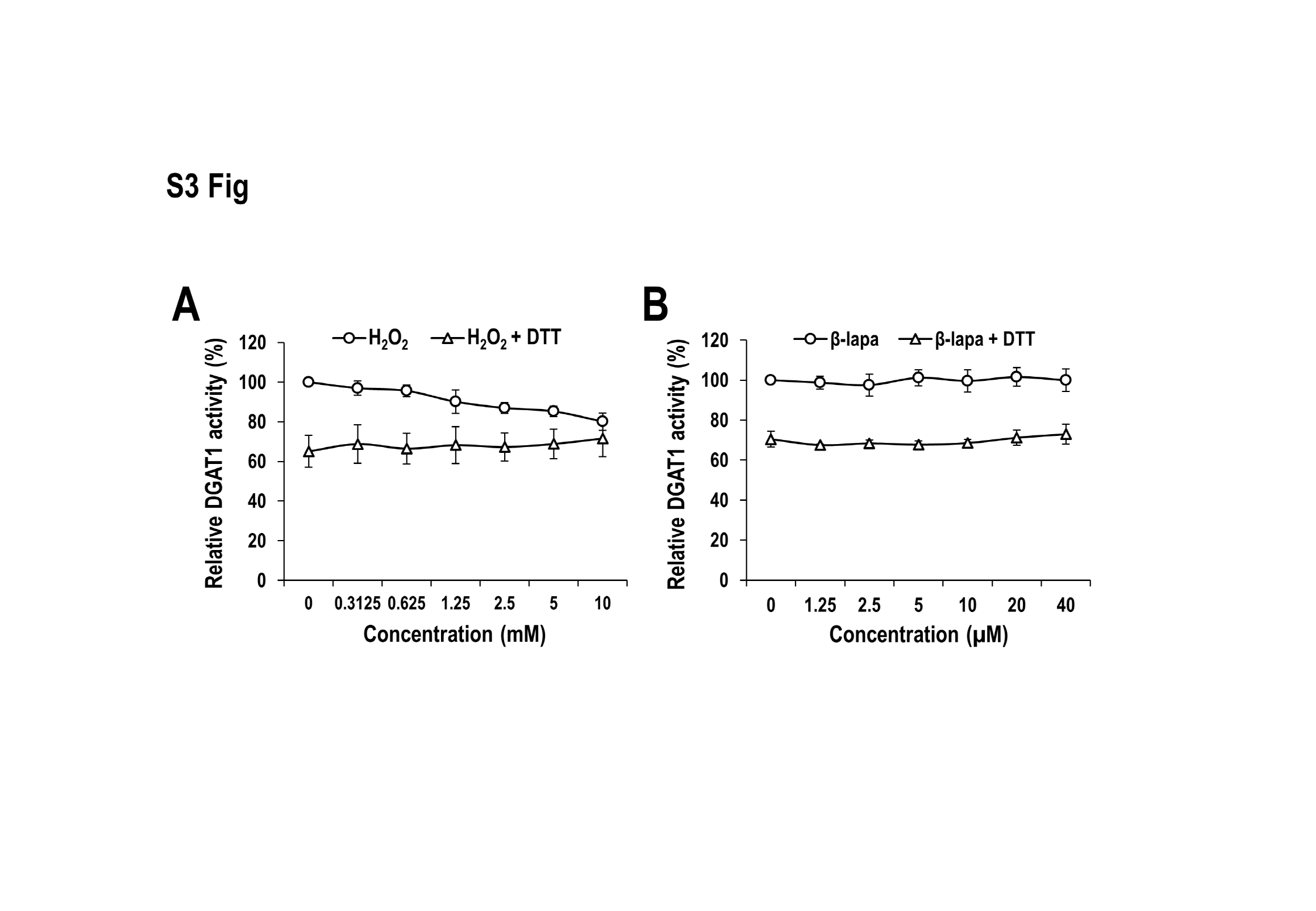

Supplement: S3 Fig — Membrane extracts from human DGAT1-overexpressing Sf9 insect cells were treated with indicated concentrations of H2O2 (A) or β-lapachone (B) in the presence or absence of 20 mM DTT. The activities of membrane extracts treated with PBS (instead of H2O2) or DMSO (instead of β-lapachone) in the absence of DTT were defined as 100%. The mean values and standard deviations were determined from four independent experiments. (TIF) [file pone.0181076.s003.TIF]

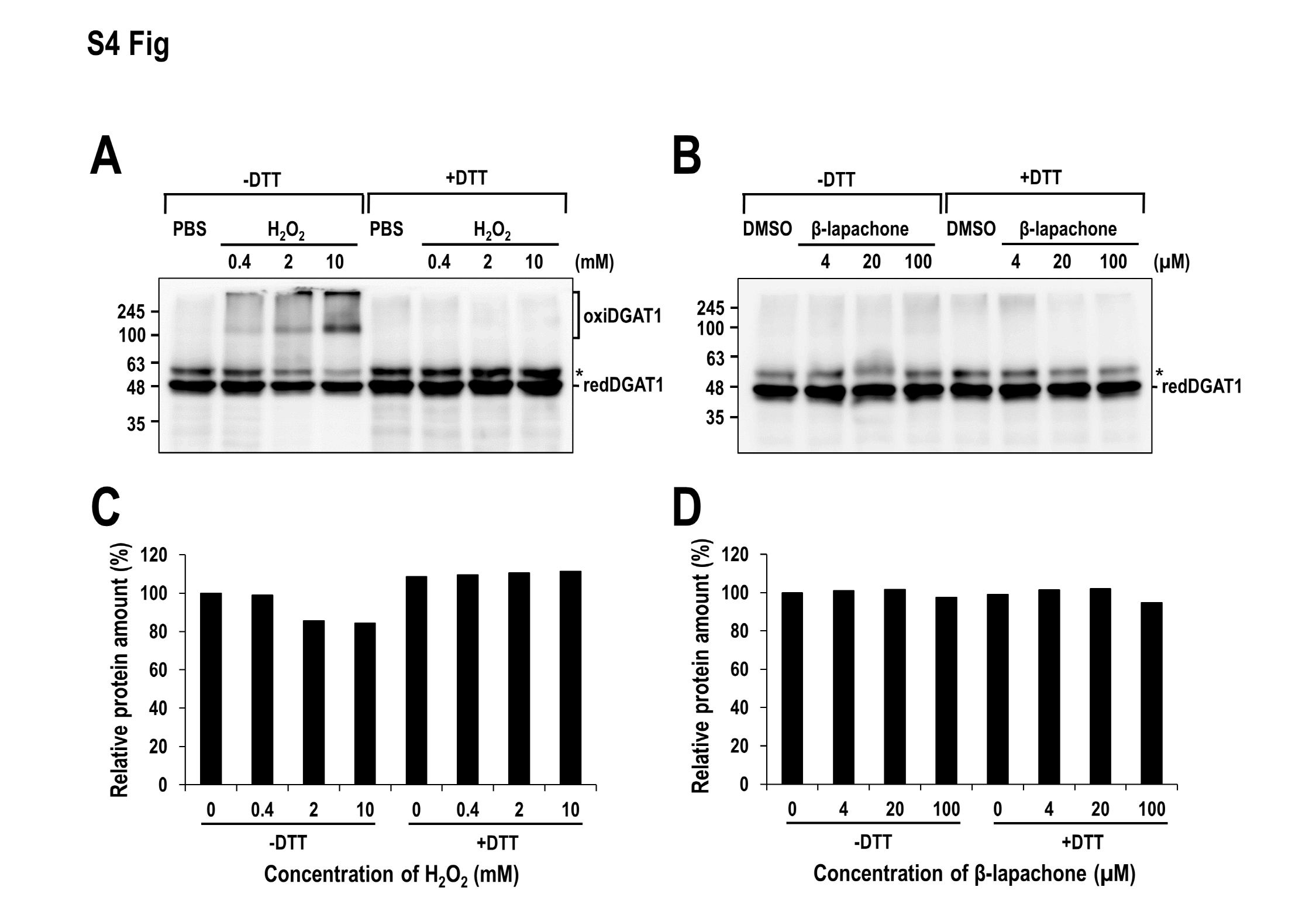

Supplement: S4 Fig — Membrane extracts from human DGAT1-overexpressing Sf9 insect cells were treated with H2O2 (A) or β-lapachone (B) in the presence or absence of 20 mM DTT and subjected to Western blot analysis using anti-DGAT1 antibody. The amount of monomeric human DGAT1 proteins presented as redDGAT1 in (A) and (B) was quantified and relative redDGAT1 protein amount were calculated by setting the values from samples treated with PBS (C) or DMSO (D) to 100%. Asterisk indicates a non-specific band. (TIF) [file pone.0181076.s004.TIF]

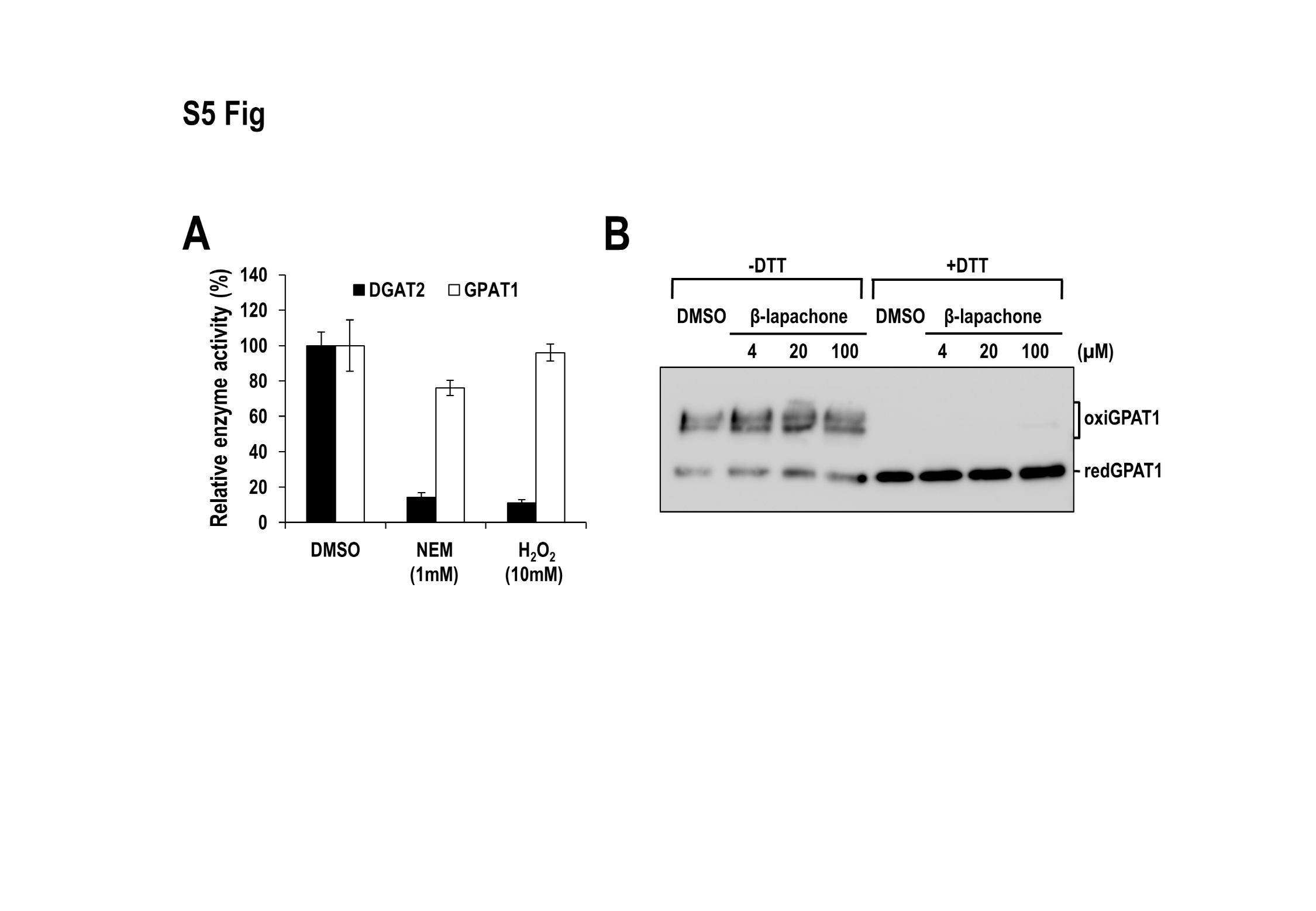

Supplement: S5 Fig — (A) The effect of NEM and H2O2 on human GPAT1 activity compared to that on human DGAT2. Membrane extracts from human DGAT2- or GPAT1-overexpressing Sf9 insect cells were treated with indicated concentrations of NEM or H2O2. Human DGAT2 and GPAT1 activities were measured by using the conventional extraction-based in vitro assays which are described in detail in the Materials and Method section. The relative enzyme activity in percentage was calculated by setting the value from DMSO-treated sample to 100%. The mean values and standard deviations were determined from three independent assays. (B) Membrane extracts from human GPAT1-overexpressing Sf9 insect cells were treated with β-lapachone in the presence or absence of 20 mM DTT and subjected to Western blot analysis using anti-GPAT1 antibody. Monomeric human GPAT1 proteins were presented as redGPAT1. (TIF) [file pone.0181076.s005.TIF]

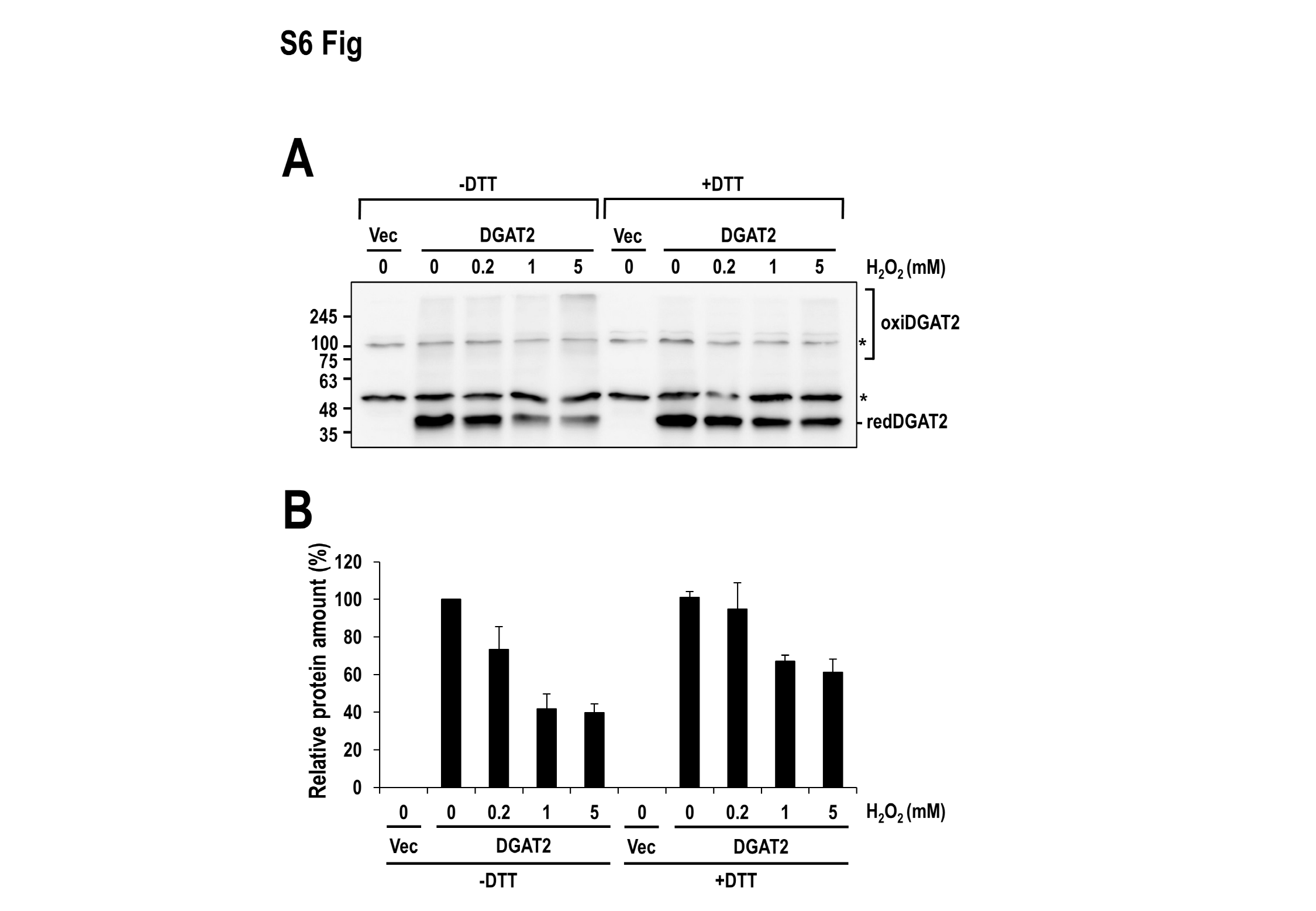

Supplement: S6 Fig — HEK293 cells were transfected with plasmid overexpressing human DGAT2 for 47 hours and further incubated with indicated concentrations of H2O2 for 1 hour. Cell extracts were harvested in a way described in Materials and Methods section and subjected to Western blot analysis using anti-DGAT2 antibody (A). The amount of monomeric human DGAT2 proteins presented as redDGAT2 in (A) was quantified and the amount of relative redDGAT2 protein was calculated by setting the values from samples treated with PBS to 100% (B). The mean values and standard deviations were determined from three independent experiments. Asterisks indicates a non-specific bands. (TIF) [file pone.0181076.s006.TIF]
